# Assessment of Knowledge and Practice Regarding Psychological First Aid Among Secondary School Students in Erbil City

**DOI:** 10.7759/cureus.64671

**Published:** 2024-07-16

**Authors:** Jawdat Mamand Akhagbaker, Mosleh Saber Kareem, Ahmed Ali Rasool, Abdulmalik F Saber, Kareem Fattah Aziz

**Affiliations:** 1 Department of Community Nursing, College of Nursing, Hawler Medical University, Erbil, IRQ; 2 Department of Psychiatric and Mental Health Nursing, College of Nursing, Hawler Medical University, Erbil, IRQ

**Keywords:** erbil city, secondary school students, practice, knowledge, psychological first aid

## Abstract

Background and aim: Secondary school students in Erbil City face various stressors and challenges that may impact their mental well-being. This study aimed to assess the knowledge and practical application of psychological first aid (PFA) among these students.

Method: This cross-sectional study was conducted from June 10th to June 25th, 2024, in six high schools in Erbil City, Iraq. Participants were selected using a purposive sampling method. Data were collected using two self-structured questionnaires, which included demographic information, a knowledge assessment, and a practical application assessment of PFA. Statistical analysis was performed using SPSS version 28 (IBM Corp., Armonk, NY). Frequency and percentage were used for categorical variables, and mean and standard deviation for quantitative variables. Ordinal regression analyses were conducted to assess the relationships between demographic variables and PFA knowledge and practice. Spearman correlation was used to determine the relationship between knowledge and practice. A p-value of less than 0.05 was considered statistically significant.

Results: A total of 412 students were enrolled in the study. The mean scores for knowledge and practice indicated fair levels, with mean scores of 7.66 ± 1.34 for knowledge and 7.01 ± 1.38 for practice. The analysis showed that 11 students (2.7%) had poor knowledge, 161 students (39.1%) had fair knowledge, and 240 students (58.2%) had good knowledge of PFA. In terms of practice, 15 students (3.6%) exhibited poor practice, 237 students (57.6%) demonstrated fair practice, and 160 students (38.8%) showed good practice of PFA. Significant associations were found between several demographic variables and PFA knowledge and practice. Males had higher knowledge scores (estimate = 1.22, 95% CI: 0.20, 2.24, P = 0.02), as did students from families with insufficient monthly income (estimate = 0.91, 95% CI: 0.10, 1.72, P = 0.03) and those residing in urban areas (estimate = 0.83, 95% CI: 0.17, 1.50, P = 0.01). For practice, the occupation of the father was significant, with unemployed fathers associated with lower practice scores (estimate = -1.08, 95% CI: -2.06, -0.09, P = 0.03).

Conclusions: The study showed that students had fair knowledge and practice of PFA. To improve these scores, it is recommended that nurses and educators develop targeted interventions and training programs. These should focus on enhancing students' understanding and practical skills in PFA, ensuring they are better equipped to handle psychological distress among their peers.

## Introduction

Psychological first aid (PFA) is an evidence-informed approach that aims to help individuals in the immediate aftermath of a crisis event, disaster, or traumatic incident [1]. Its objective is to provide compassionate, supportive, and practical assistance to people who are distressed, with the goal of promoting safety, calming, and stabilizing their emotional state [2]. By offering immediate emotional and practical support, PFA helps individuals regain a sense of control and reduces the risk of long-term psychological harm. Research indicates that timely intervention through PFA can significantly decrease the likelihood of developing post-traumatic stress disorder (PTSD) by up to 30% [3]. Furthermore, studies have shown that individuals who receive PFA are 25% less likely to experience severe depression following a traumatic event [1,4]. PFA is meant to be administered by individuals who have received appropriate training, including first responders, healthcare professionals, and members of the community [5,6].

The importance of PFA cannot be overstated as it plays a crucial role in mitigating the potentially devastating psychological impacts of traumatic events. Research indicates that without timely intervention, individuals who have experienced trauma are at a significant risk of developing mental health issues, with approximately 20% of such individuals potentially facing severe psychological challenges [7]. People who experience such events may exhibit a range of reactions, including fear, anxiety, sadness, anger, and confusion, as well as physical symptoms like headaches, nausea, and fatigue [8,9]. Without proper support and intervention, these reactions can worsen and lead to more severe psychological consequences. For example, about 8% of trauma survivors are at risk of developing PTSD [10], and the prevalence of depression among those affected by disasters can increase by up to 21% [11]. These statistics underscore the urgent need for effective mental health interventions in the aftermath of traumatic events.

Implementing PFA is particularly relevant in settings where individuals may be at an increased risk of experiencing traumatic events, such as conflict zones, areas affected by natural disasters, or communities with high rates of violence [12]. In these high-risk environments, PFA can serve as a critical component of the overall disaster response strategy, helping to lay the groundwork for long-term recovery and resilience. In these contexts, providing PFA can be a crucial first step in promoting resilience and preventing the development of long-term mental health issues [13]. Furthermore, the provision of PFA is not limited to crisis situations or traumatic events. It can also be beneficial in various other settings, such as schools, where students may face a range of stressors and challenges that can impact their mental well-being [14]. Adolescence is a particularly vulnerable period, during which individuals may undergo significant life changes, experience peer pressure and academic stress, and explore their identity [15,16]. These experiences can potentially contribute to the development of mental health issues, like anxiety and depression, which can have long-lasting effects on an individual's overall well-being and functioning [17]. By equipping secondary school students with knowledge and skills related to PFA, they can better provide initial support and assistance to their peers who may be experiencing distress or trauma [18]. This approach not only promotes a supportive and caring environment within the school community but also fosters a sense of empowerment and resilience among students. Introducing PFA concepts at an early age can also contribute to the development of positive coping mechanisms and help-seeking behaviors, which can benefit individuals throughout their lives [14]. Additionally, cognitive behavioral therapy (CBT) can be a useful intervention for these psychological disorders, much like PFA. CBT focuses on changing negative thought patterns and behaviors, offering another effective strategy for managing mental health issues and promoting long-term psychological well-being [19].

Despite the potential benefits of PFA training for secondary school students, the existing literature reveals several gaps and limitations. Firstly, many studies in this area have focused on specific populations or geographic regions, making it difficult to generalize their findings to broader contexts. Secondly, there is a lack of standardized curricula and evaluation methods for PFA training programs, which can result in inconsistencies in the implementation and assessment of these interventions. Additionally, while the immediate effects of PFA training have been explored, there is limited research on the long-term impacts and sustainability of the acquired knowledge and skills among students. Therefore, this study aims to comprehensively assess the knowledge and practices regarding PFA among secondary school students in Erbil City.

## Materials and methods

Study design, setting, and period

This study was a descriptive, cross-sectional study conducted in six high schools in Erbil City, the capital of the Kurdistan Region in Iraq, from the 10th of June 2024 to the 25th of June 2024. The study utilized a purposive sampling method to select participants. The chosen schools were Kurdistan High School, Rizgary High School, Shex Mahmud High School, Sazan High School, Hallala High School, and Shorsh High School, with a total student population of 900. The data were collected from three municipalities within Erbil City, ensuring comprehensive coverage of the area. From these, 412 students were proportionally selected for participation, ensuring a representative sample. The distribution of selected students from each school was as follows: 111 from Kurdistan High School, 49 from Rizgary High School, 41 from Shex Mahmud High School, 79 from Sazan High School, 36 from Hallala High School, and 96 from Shorsh High School.

Sample size

Given that the population of students in Erbil exceeds 150,000, we applied the infinite population formula. With the assumption of prevalence p = 0.50 (the largest sample size), a margin of error of 0.05 (5%), and α = 0.05, resulting in Zα/2 = 1.96, the smallest required sample size was calculated to be 385 samples. Due to the strong cooperation and participation of the students and staff at the selected high schools, we were able to collect data from a total of 412 samples.

Inclusion/exclusion

The study's inclusion criteria were students who agreed to participate, were functionally independent, and did not have mental disorders. The exclusion criteria included students who were not interested in participating and those who did not complete more than 95% of the questionnaire.

Study tools and data collection

The questionnaire was divided into three main parts. The first part collected demographic data, such as age group, gender, municipality, name of high school, education level of father, education level of mother, monthly income, occupation of father, occupation of mother, and residential area. The second part consisted of a self-structured questionnaire with 10 questions designed to assess students' knowledge of PFA. The third part included 10 questions that evaluated the practical application of PFA skills. The questionnaire was translated from English to Kurdish using a backward-forward method. To ensure the accuracy of the translation, it was reviewed by a psychiatrist. Data were collected by distributing the questionnaires to students who met the inclusion criteria, and each student was given 15 minutes to complete the questionnaire.

Pilot study

The study questionnaires were tested in an initial study conducted between April 12th, 2024, and May 12th, 2024. Thirty students from the general population participated in this study to assess the internal consistency and reliability of the items before they were used in the actual study. Cronbach's alpha was used to calculate the internal consistency of the items [20]. The knowledge assessment questionnaire had an overall internal reliability result of 0.91, indicating excellent reliability. On the other hand, the practice assessment questionnaire had an overall internal reliability result of 0.83, demonstrating very good reliability. These results suggest that both questionnaires have acceptable internal consistency and reliability. It is important to note that the data from this initial study were excluded from the final analysis.

Measures

Sociodemographic Characteristics

The demographic data included age, gender, municipality, education level of father, education level of mother, monthly income, occupation of father, occupation of mother, and residential area.

Knowledge Assessment Questionnaire

The second part of the self-structured questionnaire evaluates the students' knowledge regarding PFA. It consists of 10 questions designed to assess understanding of key concepts and principles of PFA. The questions include true/false and multiple-choice formats. Each correct answer is awarded one point, with a total possible score of 10 points. The scores are categorized as follows: zero to four points indicate poor knowledge, five to seven points indicate fair knowledge, and eight to 10 points indicate good knowledge. To ensure the reliability of this tool, a Cronbach's alpha coefficient was calculated [20], resulting in a value of 0.91. A pilot study for this tool was conducted over a one-month period, and the reliability was confirmed through this process.

Practice Assessment Questionnaire

The third part of the self-structured questionnaire assesses the students' practical application of PFA skills. It consists of 10 questions that evaluate students' ability to apply PFA in various scenarios. The questions include true/false and multiple-choice formats. Each correct answer is awarded one point, with a total possible score of 10 points. The scores are categorized as follows: zero to four points indicate poor practice, five to seven points indicate fair practice, and eight to 10 points indicate good practice. To ensure the reliability of this tool, a Cronbach's alpha coefficient was calculated [20], resulting in a value of 0.83. A pilot study for this tool was conducted over a one-month period, and the reliability was confirmed through this process.

Ethical approval and informed consent

This study adhered to the guidelines of the Institutional Research Ethics Board and the Declaration of Helsinki. Ethics approval was obtained from the Hawler Medical University Ethics Committee on the 9th of June, 2024, with code number 17. For students under the age of 18 years, a formal consent form was sent to their parents for signature. Only after obtaining consent from both the parents and the students were these students included in the study. For students who were 18 years or older, participation was based on their informed consent, requiring only their signature.

Statistical analysis

Data were summarized and reported with frequency and percentage for qualitative variables. Quantitative variables with a normal distribution were presented with mean and standard deviations. The adjusted association between the knowledge and practice of PFA and other confounding factors was evaluated using ordinal regression analysis. Spearman correlation was used to assess the relationship between knowledge and practice. Data analysis was performed using SPSS version 28 (IBM Corp., Armonk, NY), with significance levels considered at P < 0.05.

## Results

Demographic characteristics

A total of 412 responses were analyzed, with all participants successfully completing the questionnaire. The ages of participants ranged from 14 to 19 years, with a mean age of 16.53 ± 1.14 years. The gender distribution was nearly equal with 201 (48.8%) male and 211 (51.2%) female participants. Most students came from urban areas (360, 87.4%). Educational levels of parents varied with 170 (41.3%) of fathers and 127 (30.8%) of mothers having secondary education. The majority of families (230, 55.8%) had an insufficient monthly income. Detailed demographic data and findings are presented in Table 1.

**Table 1:** Demographic characteristics of the students. N = number; F = frequency; % = percentage; M = municipality; SD = standard deviation.

Items	Categories (n = 412)	F	%
Age (years)	14-15	63	15.3
	16-17	252	61.2
	18-19	97	23.5
	Mean ± SD	16.53 ± 1.14	
Gender	Male	201	48.8
	Female	211	51.2
Municipality	M1	188	45.6
	M2	96	23.3
	M3	128	31.1
Education of father	Illiterate	23	5.6
	Primary	125	30.3
	Secondary	170	41.3
	Diploma or bachelor	94	22.8
Education of mother	Illiterate	74	18.0
	Primary	154	37.4
	Secondary	127	30.8
	Diploma or bachelor	57	13.8
Income monthly	Insufficient	230	55.8
	Somehow sufficient	153	37.1
	Sufficient	29	7.0
Occupation of father	Employed	177	43.0
	Unemployed	23	5.6
	Retired	18	4.4
	Self-employed	194	47.1
Occupation of mother	Employed	83	20.1
	Housewife	304	73.8
	Self-employed	25	6.1
Residential area	Urban	360	87.4
	Rural	4	1.0
	Suburban	48	11.7

Knowledge and practice levels

The mean scores indicated fair knowledge and practice, with mean scores of 7.66 ± 1.34 for knowledge and 7.01 ± 1.38 for practice. Regarding knowledge levels about PFA, 240 (58.2%) of students had good knowledge while 161 (39.1%) had fair knowledge. Practical application levels showed that 237 (57.6%) of students had fair practice skills and 160 (38.8%) had good practice skills (Table 2).

**Table 2:** Distribution of knowledge and practice levels among secondary school students in Erbil City. N = 412; F = frequency; % = percentage; SD = standard deviation.

Category	Level	Frequency (n)	Percentage (%)
Knowledge levels			
	Poor	11	2.7
	Fair	161	39.1
	Good	240	58.2
	Mean ± SD	7.66 ± 1.34	
Practice levels			
	Poor	15	3.6
	Fair	237	57.6
	Good	160	38.8
	Mean ± SD	7.01 ± 1.38	

Knowledge items

The assessment of students' knowledge regarding PFA revealed several findings. A total of 211 (51.2%) correctly identified the definition of PFA. A significant majority, 315 (76.5%), understood that preparation and training can make traumatic events less stressful. A total of 334 (81.1%) recognized that common reactions to stress include headache, nausea, dizziness, fatigue, and loss of appetite. Most students, 368 (89.3%), understood that the goal of providing PFA is to create an environment of safety, connectedness, and empowerment. However, 141 (34.2%) were incorrect about linking being a key purpose of PFA, and 140 (34.0%) did not know that quietness in children can be a sign of distress. The concept of confidentiality was well understood by 385 (93.4%) of students. Additionally, 351 (85.2%) correctly identified that PFA is not counseling by professionals, and 380 (92.2%) understood the importance of finding out more about the situation and available services. Lastly, 270 (65.5%) correctly answered that telling an affected person how they should be feeling is not appropriate. Detailed results are presented in Table 3.

**Table 3:** Frequency and percentage of correct and incorrect responses to knowledge questions on psychological first aid. N = 412; F = frequency; % = percentage; PFA = psychological first aid.

Knowledge questions	Incorrect answer		Correct answer	
	F	%	F	%
Definition of PFA	201	48.8	211	51.2
Factors that make traumatic events less stressful include preparation and training.	97	23.5	315	76.5
Headache, nausea, dizziness, fatigue, and loss of appetite are common reactions to stress.	78	18.9	334	81.1
The goal of providing PFA is to create an environment of safety, connectedness, and empowerment.	44	10.7	368	89.3
Linking is one of the key purposes of PFA.	141	34.2	271	65.8
Quietness in children can be a sign of distress.	140	34.0	272	66.0
Confidentiality means not telling other people’s secrets.	27	6.6	385	93.4
PFA is not counseling by professionals.	61	14.8	351	85.2
Finding out more about the situation and available services to assist people in meeting their needs.	32	7.8	380	92.2
Telling an affected person how they should be feeling (e.g., "you should feel lucky").	142	34.5	270	65.5

Practice items

The assessment of students' practical application of PFA skills yielded varied results. Among the students, 260 (63.1%) correctly identified the importance of effective communication during times of trauma. A significant number of students (281, 68.2%) recognized that common emotional reactions can be anticipated during a disaster. The concept of active listening was well understood by 345 (83.7%) of students. However, it is worth noting that 117 (28.4%) of students incorrectly believed that forcing people to share their stories comes after ensuring safety. Furthermore, a substantial number of students (296, 71.8%) did not understand that PFA involves listening to those who wish to share their stories and emotions. On a positive note, over half of the students (255, 61.9%) correctly indicated that children may feel more secure in a school following a traumatic event. The majority of students also understood that providing immediate psychotherapy during a traumatic event is not helpful (357, 86.7%), and that offering reassurance to help people feel better is appropriate (352, 85.4%). Additionally, 312 (75.7%) of students correctly stated the importance of taking time to ensure safety before approaching a crisis scene. Finally, 319 (77.4%) of students were knowledgeable about the relaxation exercises used to reduce stress (Table 4).

**Table 4:** Frequency and percentage of correct and incorrect responses to practice questions on psychological first aid. N = 412; F = frequency; % = percentage; PFA = psychological first aid.

Practice questions	Incorrect answer		Correct answer	
	F	%	F	%
Effective communication during times of trauma includes avoiding repeating information.	152	36.9	260	63.1
Many emotional reactions that would appear unusual in a stable situation are common and can be anticipated during a disaster.	131	31.8	281	68.2
Active listening includes informing the person that "I know how you feel" and avoiding asking clarifying questions.	67	16.3	345	83.7
Forcing people to share their stories, especially personal details, comes after assuring safety.	117	28.4	295	71.6
PFA involves listening to people who wish to share their stories and emotions.	296	71.8	116	28.2
Children may feel more secure in school after a traumatic event.	157	38.1	255	61.9
During a traumatic event, it is helpful to provide immediate psychotherapy.	55	13.3	357	86.7
Giving any reassurance to help people feel better (e.g., "Your house will be rebuilt soon").	60	14.6	352	85.4
Taking time to be sure it is safe to approach the scene of a crisis event even if you must act urgently.	100	24.3	312	75.7
Which one of the following relaxation exercises is used to reduce stress?	93	22.6	319	77.4

Correlation between knowledge and practice analysis

The results of the Spearman correlation analysis revealed a positive correlation between knowledge and practice regarding PFA among secondary school students in Erbil City. Specifically, the correlation coefficient for knowledge and practice was 0.14, with a significance level of p < 0.001, indicating a statistically significant, albeit weak, positive correlation. This suggests that as students' knowledge about PFA increases, their practice of it also improves to a certain extent. For more details, please refer to Table 5.

**Table 5:** The relationship between knowledge and practice regarding psychological first aid among secondary school students in Erbil City. Note: Significance was set at P < 0.05.

Variables	Spearman	Knowledge	Practice
Knowledge	Correlation coefficient	1.00	0.14
	Sig. (2-tailed)	.	p < 0.001
	N	412	412
Practice	Correlation coefficient	0.14	1.00
	Sig. (2-tailed)	p < 0.001	.
	N	412	412

Demographic factors affecting knowledge and practice levels

The analysis of the relationship between demographic variables and knowledge and practice of PFA revealed several significant findings (Table 6). Gender showed a significant association with knowledge, where males had higher knowledge scores (estimate = 1.22, 95% CI: 0.20, 2.24, P = 0.02) compared to females. Monthly income was found to be significantly associated with knowledge. Students from families with insufficient income demonstrated higher levels of knowledge (estimate = 0.91, 95% CI: 0.10, 1.72, P = 0.03) compared to those earning more than three million. Furthermore, the urban residential area was significantly associated with higher knowledge scores (estimate = 0.83, 95% CI: 0.17, 1.50, P = 0.01) compared to suburban areas. For practice, the occupation of the father showed a significant association, with unemployed fathers linked to lower practice scores (estimate = -1.08, 95% CI: -2.06, -0.09, P = 0.03) compared to self-employed fathers.

**Table 6:** Ordinal regression of factors affecting knowledge and practice of psychological first aid among secondary school students in Erbil City (link = logit)ᵃ Statistical test: Ordinal regression analysis. Significance was set at P < 0.05. ^a^ Reference parameter. F = frequency; % = percentage; M = municipality; LB = lower bound; UB = upper bound.

Variables	Knowledge				Practice			
	Estimate	95% CI		P-value	Estimate	95% CI		P-value
		LB	UB			LB	UB	
Age								
14-15	-0.10	-1.11	0.92	0.86	0.33	-0.66	1.32	0.52
16-17	0.08	-0.73	0.89	0.84	-0.03	-0.82	0.77	0.95
18-19	0ᵃ	-	-	-	0ᵃ	-	-	-
Gender								
Male	1.22	0.20	2.24	0.02	-0.78	-1.78	0.23	0.13
Female	0ᵃ	-	-	-	0ᵃ	-	-	-
Municipality								
M1	-0.08	-3.27	3.11	0.97	-1.88	-5.65	1.90	0.33
M2	0.80	-2.48	4.08	0.64	-1.66	-5.51	2.19	0.40
M3	0ᵃ	-	-	-	0ᵃ	-	-	-
Education of father								
Illiterate	0.05	-0.98	1.08	0.93	-0.37	-1.41	0.68	0.49
Primary	-0.03	-0.71	0.66	0.94	0.02	-0.65	0.68	0.97
Secondary	-0.27	-0.88	0.34	0.39	-0.07	-0.66	0.53	0.83
Diploma or bachelor	0ᵃ	-	-	-	0ᵃ	-	-	-
Education of mother								
Illiterate	0.39	-0.51	1.29	0.40	-0.74	-1.62	0.14	0.10
Primary	-0.02	-0.83	0.80	0.97	0.20	-0.59	0.99	0.62
Secondary	-0.36	-1.13	0.42	0.37	0.12	-0.63	0.86	0.76
Diploma or bachelor	0ᵃ	-	-	-	0ᵃ	-	-	-
Income monthly								
Insufficient	0.91	0.10	1.72	0.03	0.20	-0.62	1.02	0.63
Somehow sufficient	0.80	-0.02	1.63	0.06	0.35	-0.49	1.18	0.41
Sufficient	0ᵃ	-	-	-	0ᵃ	-	-	-
Occupation of father								
Employed	-0.02	-0.48	0.45	0.95	-0.05	-0.50	0.41	0.85
Unemployed	-0.71	-1.63	0.22	0.13	-1.08	-2.06	-0.09	0.03
Retired	0.01	-1.10	1.12	0.99	-0.23	-1.30	0.84	0.67
Self-employed	0ᵃ	-	-	-	0ᵃ	-	-	-
Occupation of mother								
Employed	-0.00	-1.00	0.99	0.99	-0.64	-1.59	0.31	0.19
Housewife	-0.35	-1.27	0.57	0.46	-0.32	-1.20	0.56	0.47
Self-employed	0ᵃ	-	-	-	0ᵃ	-	-	-
Residential area								
Urban	0.83	0.17	1.50	0.01	-0.05	-0.72	0.62	0.88
Rural	0.76	-1.44	2.95	0.50	-0.42	-2.60	1.76	0.71
Suburban	0ᵃ	-	-	-	0ᵃ	-	-	-

## Discussion

The present study aimed to assess the knowledge and practice of secondary school students in Erbil City regarding PFA. Overall, the results indicated that the students had a fair level of knowledge and practice when it comes to PFA. Additionally, the study found significant associations between certain demographic factors and students' levels of knowledge and practice. Male students demonstrated higher knowledge levels compared to female students. Students from families with insufficient monthly income and those living in urban areas also showed greater knowledge of PFA, while students whose fathers were unemployed exhibited lower practice levels of PFA.

The PFA is an evidence-informed approach designed to assist individuals immediately after a traumatic event, crisis, or disaster [1]. It involves providing humane, supportive, and practical assistance to address immediate needs and facilitate adaptive coping mechanisms [2]. Introducing PFA concepts and skills to students can empower them to respond effectively in emergency situations and support their peers or community members during times of distress. This empowerment is crucial for fostering a resilient and responsive youth population capable of mitigating the psychological impacts of trauma. Recognizing the importance of equipping young individuals with these critical skills, the researchers aimed to assess the levels of knowledge and practice regarding PFA among secondary school students in Erbil City. By understanding their current capabilities, targeted educational programs can be developed to enhance their preparedness and effectiveness in real-life situations. Additionally, integrating PFA training into the school curriculum and conducting regular workshops can ensure that students continually develop and refine their skills.

The study's demographic breakdown, featuring a nearly equal gender distribution and a predominantly urban sample, offers a representative snapshot of the secondary school student population in Erbil City. The diverse educational levels of parents and varying family income levels provide further context to the socioeconomic backgrounds of the participants, potentially influencing their access to mental health education and resources. This demographic diversity aligns with findings from other studies, which have identified socioeconomic factors as significant barriers or facilitators to mental health literacy and help-seeking behaviors [21,22]. Understanding these socioeconomic influences is crucial for developing targeted interventions that address the specific needs of different student groups and improve overall mental health outcomes.

The assessment of students' knowledge regarding PFA revealed both strengths and areas for improvement. It is encouraging that a significant number of students correctly identified key concepts, such as the definition of PFA, the impact of preparation and training, and common stress reactions. This suggests a basic understanding of the purpose and relevance of PFA among the participants. However, there are misconceptions regarding the purposes of PFA and signs of distress in children, which highlights the need for more comprehensive education in these specific areas. Similar knowledge gaps have been observed in other studies conducted among various populations, including healthcare professionals and community members [6,23]. This emphasizes the importance of tailored educational interventions.

The results regarding the practical application of PFA skills also showed that the participants had a fair level of practice. Although a significant number of students demonstrated fair practice skills, it is encouraging to see a notable number of students achieving a good level of practice. Effective communication and the ability to identify common emotional reactions during disasters are essential aspects of PFA. The findings of this study suggest that many students possess these competencies. This is consistent with previous research that has emphasized the significance of communication skills and emotional intelligence in implementing PFA [24]. However, there are concerns regarding the misconceptions surrounding the idea of forcing individuals to share their stories and the role of PFA in listening to those who want to express their emotions. These misunderstandings could potentially result in inappropriate or harmful responses in real-life situations, underscoring the significance of addressing these knowledge and practice gaps. Similar challenges have been reported in other contexts, where cultural beliefs or societal norms might influence perceptions and practices related to mental health support [22,25]. On a positive note, many students recognize the importance of safety, immediate psychotherapy, and relaxation exercises, which are essential elements of PFA.

The analysis of the relationship between demographic variables and PFA knowledge and practice provided additional insights. The higher knowledge scores observed among male students align with previous research suggesting gender differences in mental health literacy [26]. However, another study has reported contradictory findings or no significant gender differences [27], indicating that sociocultural factors and educational experiences may play a role in shaping these disparities. In the context of Erbil City and the Kurdistan Region, cultural factors such as traditional gender roles, family expectations, and varying access to educational opportunities might influence these differences [28]. It is essential to explore the underlying factors contributing to these gender differences and address them through targeted educational interventions tailored to the specific needs and contexts of different genders. The positive association between higher monthly income, urban residence, and better knowledge levels is consistent with existing literature linking socioeconomic status and access to education with improved mental health literacy [29]. However, it is important to note that some studies have found more complex interactions between socioeconomic factors and mental health knowledge [29,30]. Ensuring equitable access to mental health education and resources across diverse socioeconomic backgrounds is crucial for promoting widespread PFA awareness and preparedness.

The finding that the occupation of the father significantly influenced students' practice skills is noteworthy. Unemployed fathers were associated with lower practice scores compared to self-employed fathers. This relationship may be influenced by various factors, including parental involvement, role modeling, and the potential impact of employment status on family dynamics and resource availability [31]. However, caution is necessary when interpreting this finding. The relationship between parental employment and children's mental health literacy may be mediated by other factors, such as parental education levels, family support systems, and cultural norms [32]. Further research is needed to explore the underlying mechanisms that influence PFA knowledge and practice among students from diverse family backgrounds. Future studies should focus on developing targeted interventions tailored to the unique socioeconomic and educational needs of these students. By addressing these factors, we can enhance the effectiveness of PFA training and support for all students, ensuring better mental health outcomes.

Significance and limitations of the study

The study utilized a comprehensive and well-structured questionnaire that was rigorously tested for reliability through a pilot study, ensuring accurate and consistent data collection. With a large sample size of 412 students from various high schools across three municipalities, the study provides a representative snapshot of the student population in Erbil City. The cross-sectional design allowed for the identification of significant associations between demographic variables and PFA knowledge and practice, offering valuable insights for targeted interventions. However, a key limitation of the study is that its findings are specific to the six high schools in Erbil City and may not be generalizable to other regions of Iraq or different populations. Additionally, the study did not account for potential cultural differences within different regions, which might influence the generalizability of the findings.

## Conclusions

The study showed that students had fair knowledge and practice of PFA. To improve these scores, it is recommended that nurses and educators develop targeted interventions and training programs. These programs should focus on enhancing students' understanding and practical skills in PFA. By doing so, students will be better equipped to handle psychological distress among their peers. Improved training will help create a more supportive and resilient school environment. Additionally, the government should invest in mental health education and resources in schools. Policies should be implemented to ensure regular training and updates for students and staff on PFA techniques.
